# Engineered Exosomes Complexed with Botulinum Toxin Type A for Enhanced Anti-Aging Effects on Skin

**DOI:** 10.3390/biology14081040

**Published:** 2025-08-13

**Authors:** Yaru Wang, Kunju Wang, Xinyu Ben, Mengsi Tian, Xinyu Liu, Zaihong Li, Panli Ni, Qibing Liu, Zhijian Ma, Xinan Yi, Qingyun Guo

**Affiliations:** 1 Key Laboratory of Tropical Translational Medicine of Ministry of Education & Key Laboratory of Brain Science Research Transformation in Tropical Environment of Hainan Province, School of Basic Medicine and Life Sciences, Hainan Medical University, Haikou 571199, China; hy0206225@muhn.edu.cn (Y.W.); hy0206079@muhn.edu.cn (K.W.); benxinyu@muhn.edu.cn (X.B.); m17600838331@163.com (M.T.); enc82126@gmail.com (X.L.); hy0206180@muhn.edu.cn (P.N.); hy0207043@muhn.edu.cn (Q.L.); hy0108003@muhn.edu.cn (Z.M.); 2 Department of Human Anatomy, School of Basic Medicine and Life Sciences, Hainan Medical University, Haikou 571199, China; 3 Hainan General Hospital, Hainan Affiliated Hospital of Hainan Medical University, Haikou 570311, China; 4 Department of Ultrasound, Hainan Women and Children’s Medical Center, Haikou 570100, China; 19943343394@163.com; 5 Engineering Research Center of Tropical Medicine Innovation and Transformation, Ministry of Education, Hainan Medical University, Haikou 570102, China

**Keywords:** adipose stem cells, exosomes, botulinum toxin type A, synaptic vesicle glycoprotein 2C, cellular senescence

## Abstract

Skin aging is commonly characterized by increased wrinkles, loss of elasticity, and hyperpigmentation, significantly affecting personal appearance and quality of life. Although botulinum toxin type A (BTX-A) has been widely applied in cosmetic anti-wrinkle treatments, its intrinsic cytotoxicity limits broader clinical applications. In this study, we developed a novel exosome-based BTX-A composite delivery system designed to synergize the anti-aging properties of exosomes with the wrinkle-reducing effects of BTX-A while reducing toxicity. Human adipose-derived mesenchymal stem cells were genetically modified via lentiviral transduction to overexpress Synaptic Vesicle Glycoprotein 2C (SV2C), the receptor of BTX-A, thereby producing SV2C-enriched functionalized exosomes (EXO^SV2C^). These exosomes (2.0 × 10^7^ particles/mL) were incubated with BTX-A (3 U/mL) to generate the EXO^SV2C^-BTX-A complex. In vitro, EXO^SV2C^-BTX-A significantly promoted the proliferation and migration of human dermal fibroblasts and effectively alleviated D-galactose (D-gal)-induced cellular senescence and collagen type I loss. These effects were superior to those observed with either BTX-A or exosomes alone. In vivo, intradermal injection of EXO^SV2C^-BTX-A for 28 days markedly suppressed D-gal-induced skin aging in 8-week-old male KM mice, as evidenced by reduced malondialdehyde levels in dermal tissue, enhanced collagen type I expression, and preserved skin structure. Notably, the composite exhibited significantly lower toxicity compared to free BTX-A. Collectively, these findings highlight EXO^SV2C^-BTX-A as a promising exosome-mediated BTX-A delivery platform with enhanced anti-aging efficacy and improved biocompatibility, offering a potential therapeutic strategy for skin rejuvenation.

## Simple Summary

As people age, their skin naturally becomes wrinkled, less elastic, and more prone to damage. Botulinum toxin type A (BTX-A) is widely used in cosmetic treatments to reduce wrinkles, but it has toxic side effects that can harm healthy skin cells. In this study, we developed a new and safer method for using BTX-A by attaching it to tiny natural particles called exosomes, which are produced by stem cells. These exosomes were specially designed to carry a protein that helps them bind BTX-A more effectively and deliver it into skin cells. We found that this exosome–BTX-A combination not only reduced the signs of skin aging more effectively than BTX-A or exosomes alone, but also caused significantly less damage to cells. In both laboratory and animal experiments, the treatment improved skin structure, boosted collagen production, and lowered harmful stress markers in the skin. This research presents a promising new approach to safer and more effective anti-aging skin therapies.

## 1. Introduction

Skin, the largest organ of the human body, serves as the primary barrier protecting against external environmental insults [1]. With advancing age, the skin progressively exhibits signs of aging, such as increased wrinkles, skin laxity, and impaired cellular functions, significantly affecting an individual’s appearance and quality of life [2,3]. Therefore, identifying effective anti-aging strategies is of substantial clinical relevance and social importance. Since its initial report in 1994 for reducing facial wrinkles [4], botulinum toxin type A (BTX-A) has gained widespread acceptance and extensive use in aesthetic medicine, particularly in the treatment of glabellar lines, facial erythema, and other dermatological conditions [5,6,7].

BTX-A, a neurotoxin produced by Clostridium botulinum, exerts its effects by specifically binding to synaptic vesicle glycoprotein 2C (SV2C) and ganglioside GT1b, thereby inhibiting acetylcholine release at neuromuscular junctions. This mechanism results in muscle relaxation, thereby reducing wrinkles and achieving cosmetic benefits [8,9]. In addition to its aesthetic applications, BTX-A has been clinically used for over 150 years to treat various medical conditions, including strabismus, facial spasms, and chronic migraines [10,11,12,13]. Despite its therapeutic efficacy, BTX-A exhibits considerable toxicity, with a lethal dose of approximately 1 ng/kg, even at extremely low concentrations [14,15]. Consequently, reducing the inherent toxicity of BTX-A and enhancing its clinical safety have become important research focuses.

In recent years, exosomes have garnered significant attention as highly efficient natural drug carriers. Exosomes are nano-sized (approximately 30–200 nm in diameter), bilayer membrane-bound vesicles secreted by various cells, containing a variety of biologically active macromolecules and small molecules, thus serving as essential intercellular signaling vehicles [16,17,18,19]. Numerous studies have demonstrated that bioactive substances susceptible to degradation or inactivation can be safely and effectively delivered to target cells under the protection provided by exosomes. Hence, the application of exosomes for delivering RNA and small-molecule therapeutics has exhibited substantial clinical potential [20,21]. Moreover, exosomes derived from mesenchymal stem cells (MSCs) have demonstrated significant anti-aging effects by modulating the functions of epidermal and fibroblast cells [22]. MSC-derived exosomes effectively suppress inflammatory responses, accelerate epithelial regeneration and angiogenesis, promote wound healing [23], and alleviate ultraviolet-B-induced photoaging [24]. Our previous studies have further indicated that the combined application of exosomes and BTX-A is more effective than BTX-A alone in improving skin scarring conditions [25].

This study aims to develop a novel therapeutic strategy utilizing engineered exosomes to deliver BTX-A effectively. Human adipose-derived mesenchymal stem cells (ADMSCs), chosen for their accessibility, abundance, and low ethical risk, were transfected using lentiviral vectors to overexpress SV2C, the receptor for BTX-A, resulting in SV2C-enriched engineered exosomes (EXO^SV2C^). Subsequently, EXO^SV2C^ was co-incubated with BTX-A to form EXO^SV2C^-BTX-A complexes. This approach aims to harness the synergistic anti-aging properties of exosomes and the wrinkle-reducing capabilities of BTX-A, while significantly mitigating the cytotoxicity associated with BTX-A. The efficacy and safety of this delivery system were systematically evaluated through both in vitro cellular assays and in vivo mouse models. Overall, this study proposes a safe, effective, and clinically promising method for anti-aging skin therapy.

## 2. Materials and Methods

### 2.1. Cell Culture

HEK293T cells (CL-0005, Procell Life Science & Technology, Wuhan, China) and human skin fibroblasts (HFF-1; BFN60808577, Shanghai Yubo Biotechnology Co., Ltd., Shanghai, China) were cultured in high-glucose Dulbecco’s Modified Eagle Medium (DMEM; BL304A, Biosharp, Shanghai, China) supplemented with 10% fetal bovine serum (FBS; 12664025C, Gibco, Grand Island, NY, USA) and 1% penicillin–streptomycin (BL505A, Biosharp, China). Human adipose-derived mesenchymal stem cells (ADMSCs) were derived from a liposuction specimen of a 30-year-old healthy woman. After resting under sterile conditions for 30 min to allow stratification, 6 mL of middle-layer adipose tissue was mixed with 2 mL of phosphate-buffered saline (PBS) and 2.5 mg of type I collagenase (BS163, Biosharp, China). The mixture was then digested at 37 °C with shaking at 120 rpm for 60 min. The digestion was terminated by adding 2 mL low-glucose DMEM (BL305A, Biosharp, China), followed by centrifugation at 247× *g* for 10 min. The cell pellet was resuspended and filtered through a 70 μm strainer. The filtrate was adjusted to 4 mL and transferred into a T25 culture flask, which was then incubated at 37 °C in a 5% CO_2_ atmosphere. Medium was replaced after 24 h. Cells were passaged to P1 after 48 h and expanded to P2. Flow cytometry was used to characterize P3 ADMSCs. Following trypsinization (BL512A, Biosharp, China), 5 × 10^4^ cells per tube were incubated with antibodies against CD19 (11-0199-42, Thermo Fisher Scientific, Waltham, MA, USA), CD105 (17-1057-42, Thermo Fisher Scientific), CD44 (17-0441-82, Thermo Fisher Scientific), and CD11b (11-0118-42, Thermo Fisher Scientific) at 37 °C for 1 h and analyzed using an FACSCalibur flow cytometer (Becton Dickinson, Franklin Lakes, NJ, USA).

### 2.2. Plasmid Construction

The SV2C gene was amplified by polymerase chain reaction (PCR) using specific primers: forward primer 5′-AGGTCGACTCTAGAGGATCCCGCCACCATGGAAGACTCTTACAAGGATAG-3′ and reverse primer 5′-TCCTTGTAGTCCATACCCATCAGAACCTGGGTTCGTGTGTGTC-3′. The amplicon was cloned into the GV492 vector (GeneChem, Shanghai, China) between BamHI and AgeI restriction sites to generate the GV492-SV2C expression plasmid. The final construct was confirmed by sequencing and stored at −80 °C.

### 2.3. Lentiviral Packaging and Transduction

Lentivirus was produced according to a previously described method [20]. First, 20 µg of GV492-SV2C, 15 µg of pHelper 1.0, 10 µg of pHelper 2.0, and 1 mL of transfection reagent were mixed and incubated for 15 min at room temperature before being added to HEK293T cells. After 6 h, the medium was replaced, and cells were cultured for an additional 72 h. The supernatant was collected by centrifugation at 4200× *g* for 10 min, filtered through a 0.45 µm membrane, and concentrated by ultrafiltration at 5145 g, 4 °C, for 2 h. After a second centrifugation at 2058× *g* for 5 min, the virus was aliquoted and stored at −80 °C. Viral titer was determined using fluorescence detection. ADMSCs were infected with the lentivirus for 72 h, then selected with 2 mL medium containing 6 µg/mL puromycin. After one day, the medium was changed, and the cells were cultured for two passages. SV2C expression was assessed by fluorescence microscopy, Western blot, and immunofluorescence. For BTX-A binding analysis, 100 µL of 3 U/mL botulinum toxin A (BTX-A; HengLi^®^, Lanzhou Bioproduction Institute, Lanzhou, China) was added to the SV2C-overexpressing ADMSCs, and BTX-A binding was evaluated by immunofluorescence.

### 2.4. Quantitative PCR (qPCR)

Total RNA was extracted using TRIzol reagent (15596026CN, Invitrogen, Waltham, MA, USA) and quantified with a NanoDrop-2000 spectrophotometer (Thermo Fisher Scientific). First-strand cDNA was synthesized from total RNA using the Hifair 1st Strand cDNA Synthesis SuperMix (11120ES60, Yeasen Biotech, Shanghai, China). qPCR was conducted using Hieff qPCR SYBR Green Premix (11202ES08, Yeasen Biotech, China) with the following primers: β-actin forward 5′-GGCGGCACCACCATGTACCCT-3′ and reverse 5′-AGGGGGCCGGACTCGTCATACT-3′; SV2C forward 5′-CAAGACGGGATGTCAGATTACC-3′ and reverse 5′-AAAGCACCCATAGAGCCACCTA-3′. PCR conditions were as follows: pre-denaturation at 94 °C for 3 min, followed by 22 cycles of 94 °C for 30 s, 55 °C for 30 s, and 72 °C for 30 s, with a final extension at 72 °C for 5 min.

### 2.5. Exosome Isolation and Characterization

Exosomes were isolated from the culture supernatant of ADMSCs using differential ultracentrifugation as described previously [25]. ADMSCs were cultured in serum-free medium (DMEM/F12, Biosharp) for 72 h. The collected supernatant was sequentially centrifuged at 300× *g*, 2000× *g*, and 10,000× *g* for 10 min each at 4 °C to remove cells and debris. The resulting supernatant was filtered through a 0.22 µm microporous membrane. The filtrate was concentrated by centrifugation at 5000× *g* for 30 min and then subjected to ultracentrifugation at 100,000× *g* for 70 min. The pellet was resuspended in sterile PBS and stored at −80 °C. For morphological analysis, transmission electron microscopy (TEM) was performed. Exosomes were centrifuged at 2470× *g* for 10 min at 4 °C, and the supernatant was diluted at 1:10,000. A drop of diluted exosome solution was applied vertically onto a copper grid and air-dried under a heat lamp. The grid was then stained with 1% phosphotungstic acid for 70 s and dried again before observation under 60 kV using an HT-7800 electron microscope (Hitachi, Tokyo, Japan). Particle size and concentration were measured using a ZetaView^®^ PMX120 nanoparticle tracking analyzer (Particle Metrix, Meerbusch, Germany). To confirm the expression of exosomal surface marker CD63 and its interaction with BTX-A, flow cytometry analysis was performed. Polystyrene microspheres (Microspheres, ThermoFisher, Waltham, MA, USA) were used as capture carriers. FITC-conjugated anti-CD63 antibody (ab18235, Abcam, Cambridge, UK) was bound to biotinylated microspheres, allowing specific binding of CD63 on the exosomal surface. To detect surface-bound BTX-A, PE-conjugated anti-BTX-A antibody (ab252711, Abcam) was prepared using the Lightning-Link PE/R-Phycoerythrin Conjugation Kit (ab102918, Abcam). After staining in the dark, fluorescence signals from the FITC and PE channels were measured using a flow cytometer, allowing for the evaluation of exosome capture efficiency and verification of BTX-A binding. This method is protected by a Chinese national invention patent CN116165376A. Purified exosomes were labeled with red fluorescent dye PKH26 (MINI26-1KT, Sigma-Aldrich, St. Louis, MO, USA). The labeling reaction was terminated by adding an equal volume of exosome-free fetal bovine serum, followed by ultracentrifugation to remove excess unbound PKH26 dye and re-isolate the labeled exosome.

### 2.6. Cell Senescence Model Induction

Human skin fibroblast cells (HFF-1) were cultured in high-glucose Dulbecco’s Modified Eagle Medium (DMEM; BL304A, Biosharp) supplemented with 10% fetal bovine serum. When cell confluence reached approximately 80%, the cells were trypsinized and seeded into 6-well plates at a density of 3 × 10^5^ cells per well. Cellular senescence was induced using D-galactose at a final concentration of 40 mg/mL for 48 h. After induction, the medium was replaced with serum-free DMEM. Cells were treated according to experimental grouping as follows: 100 μL of 3 U/mL BTX-A (BTX-A group), 2.0 × 10^7^ particles/mL exosomes (EXO group), or an equivalent dose of the EXO^SV2C^-BTX-A complex (2.0 × 10^7^ particles/mL containing 3 U/mL BTX-A). Control wells received equal volumes of PBS. Cells were cultured for an additional 24 h before analysis. To ensure consistency in exosome dosage, exosome concentrations were quantified prior to treatment using nanoparticle tracking analysis, and appropriate dilutions were made to standardize the number of exosomal particles between the EXO and EXO^SV2C^-BTX-A groups.

### 2.7. Cell Viability Assay

To assess the effects of different treatments on HFF-1 cell viability, cells were seeded into 96-well plates and randomly divided into four groups: PBS, EXO, BTX-A, and EXOSV2C-BTX-A. Upon cell attachment, the culture medium was replaced with serum-free DMEM. Each group received 10 μL of 3 U/mL BTX-A solution, 2.0 × 10^7^ particles/mL exosome suspension, or an equivalent dose of the EXO-BTX-A complex; PBS was added to the control group. At 6, 12, 24, and 48 h post-treatment, 10 μL of CCK-8 reagent (C0038, Beyotime, Shanghai, China) was added to each well. Plates were incubated at 37 °C for 2 h, and absorbance was measured at 450 nm using a microplate reader (Synergy HTX, BioTek, Winooski, VT, USA) to reflect cellular metabolic activity.

### 2.8. Scratch Wound Healing Assay

Sterile 1000 μL pipette tips were used to create a vertical scratch at the center of the cell monolayer under a biosafety cabinet. Detached cells were gently washed away with PBS, and the medium was replaced with serum-free DMEM containing 100 μL of 3 U/mL BTX-A, 2.0 × 10^7^ particles/mL exosomes, or an equivalent amount of the EXO-BTX-A complex. The control group received an equal volume of PBS. Images of the scratch area were captured at 0, 12, and 24 h. ImageJ software (version 1.53; NIH, Bethesda, MD, USA) was used to calculate the wound closure rate as a measure of cell migration: Migration area (%) = (S_0_ − S_n_)/S_0_ × 100%, where S_0_ is the initial scratch area and S_n_ is the scratch area at time n.

### 2.9. Mouse Skin Aging Model

An in vivo model of skin aging was established according to a previously published method [26,27]. Sixty 8-week-old male Kunming mice were housed at 25 °C with a 12-h light/dark cycle and given free access to food and water for a 2-week acclimation period. Mice were randomly divided into five groups (*n* = 12 per group): normal control (untreated), D-galactose only, D-galactose + EXO, D-galactose + BTX-A, and D-galactose + EXO^SV2C^-BTX-A. Except for the control group, all mice received daily subcutaneous injections of D-galactose (1000 mg/kg, B21893, YuanYe Bio, Shanghai, China) into the dorsal skin for 42 consecutive days to induce aging. Starting from Day 15, mice in the treatment groups received daily subcutaneous injections of one of the following (100 μL per injection) for 28 days: exosome (2.0 × 10^7^ particles/mL), BTX-A (3 U/mL), EXO^SV2C^-BTX-A complex (2.0 × 10^7^ particles/mL, 3 U/mL BTX-A). On Day 42, mice were euthanized under anesthesia, and dorsal skin tissues were collected from the injection sites for histological and molecular biological analyses. Blood samples were also collected for hematological and serum biochemical testing. To ensure consistent exosome dosing, the particle concentration of exosomes was quantified using nanoparticle tracking analysis prior to administration, and the dosage was adjusted accordingly to ensure equal exosome amounts between the EXO and EXO^SV2C^-BTX-A groups. All animal procedures were approved by the Animal Care and Use Committee of Hainan Medical University (Approval No. HYLL-2023-026, Date: 24 February 2023).

### 2.10. Determination of Mouse Lethal Dose

To evaluate the toxicity differences between BTX-A and the EXO^SV2C^-BTX-A complex, a two-stage dose-escalation experiment was performed. In the first stage, BTX-A was administered via intraperitoneal injection at doses of 1, 2, 4, 8, 16, 32, 64, and 128 U/kg. For each dose, mice were randomly divided into two groups: BTX-A and EXO-BTX-A (2.0 × 10^7^ particles/mL exosomes), with 10 mice in each group. All mice received a single intraperitoneal injection and were monitored continuously for 4 days for signs of toxicity and mortality. Mortality rates were recorded to estimate the 0% lethal dose (LD_0_) and 100% lethal dose (LD_100_). In the second stage, doses were refined within the LD_0_–LD_100_ range and set at 32, 40, 44, 48, 52, 56, 64, 68, and 72 U/kg, with 10 mice per dose group. The same treatment and observation procedure was followed. The number of deaths within 4 days was recorded, and toxicological parameters were analyzed using the BL-420 biological function experimental system (Chengdu Taimeng Technology Ltd., Chengdu, China). LD_50_ and the 95% confidence interval (CI) were calculated to evaluate whether exosome encapsulation could mitigate BTX-A toxicity. Additionally, after 4 days of acute toxicity treatment, mice from the EXO group (2.0 × 10^7^ particles/mL), BTX-A group (at LD_50_ dose), and EXO^SV2C^-BTX-A group (at LD_50_ dose) were sacrificed for tissue analysis. Lung, liver, and kidney tissues were collected for morphological evaluation, and blood samples were obtained for complete blood count and serum biochemical analysis to assess systemic toxicity differences among the groups further.

### 2.11. Malondialdehyde Assay

To evaluate oxidative stress injury in mouse dorsal skin tissue, malondialdehyde (MDA) levels were measured using a commercial assay kit (A003-1, Nanjing Jiancheng Bioengineering Institute, Nanjing, China). Fresh tissue samples were homogenized according to the kit instructions, and protein concentration was determined using the bicinchoninic acid (BCA) method. MDA content was normalized to total protein. Absorbance at 532 nm was measured using a microplate reader, and MDA concentrations were calculated based on a standard curve and expressed as nmol/mg protein.

### 2.12. Histological Evaluation

To assess histological structure and collagen deposition in skin, tissue sections were prepared and stained as previously described [25]. Fresh dorsal skin samples were collected at the end of the experiment and fixed in 4% paraformaldehyde at 4 °C for 24 h. Tissues were processed via standard dehydration, paraffin embedding, and sectioning at a thickness of 5 μm. Hematoxylin and eosin (H&E) staining was performed according to the manufacturer’s instructions (C0105S, Beyotime, China) to evaluate tissue architecture and inflammatory infiltration. Fontana-Masson Stain (ab150669, Abcam) was used to assess collagen fiber content and distribution. Images were captured using an inverted microscope (CKX53, OLYMPUS, Tokyo, Japan) for qualitative and quantitative analyses.

### 2.13. Western Blot Analysis

Western blot analysis was conducted as described previously [28]. To detect the expression levels of target proteins, cells were lysed on ice for 5 min using RIPA buffer supplemented with phenylmethylsulfonyl fluoride (PMSF). Supernatants were collected by centrifugation, and protein concentrations were measured using a BCA protein assay kit (20201ES, Yeasen, China). Equal amounts of protein were denatured at 100 °C for 8 min, separated on 5% stacking and 12% separating SDS-PAGE gels, and transferred onto nitrocellulose (NC) membranes. After blocking with 5% bovine serum albumin at room temperature for 1 h, membranes were incubated overnight with primary antibodies at 4 °C, followed by incubation with horseradish peroxidase (HRP)-conjugated secondary antibodies for 1 h at room temperature. Protein bands were visualized using the Tannon 5200Multi chemiluminescence imaging system (Tannon, Shanghai, China). The primary antibodies used were rabbit anti-SV2C (ab33892, Abcam, 1:1000), rabbit anti-Collagen I (AF7001, Affinity Biosciences, Cincinnati, OH, USA; 1:800), and rabbit anti-β-tubulin (ab18207, Abcam, 1:800). The secondary antibody was HRP-conjugated goat anti-rabbit IgG (BL003A, Biosharp, 1:20,000). β-Tubulin served as the internal control.

### 2.14. Immunofluorescence Staining and SA-β-Galactosidase Assay

Immunofluorescence staining was used to detect SV2C and Collagen I expression, following previously established protocols [29]. Once cells reached 80% confluence, the culture media were discarded, and the cells were washed three times with PBS. They were then fixed in 4% paraformaldehyde for 30 min and blocked at room temperature for 1 h. Cells were then incubated overnight at 4 °C with primary antibodies. After rewarming for 30 min the next day, cells were incubated with fluorescent secondary antibodies for 90 min at room temperature in the dark. The secondary antibodies included donkey anti-rabbit IgG (ab150076, Abcam) and goat anti-rabbit IgG (ab150073, Abcam). After three washes, nuclei were counterstained using DAPI-containing antifade mounting medium (R20889, YuanYe Bio, China). Images were captured using an inverted fluorescence microscope (IX73, Olympus, Tokyo, Japan). To evaluate cellular senescence, senescence-associated β-galactosidase (SA-β-gal) staining was performed. Fibroblasts cultured in 6-well plates were washed twice with PBS and stained according to the manufacturer’s instructions (C0602, Beyotime, China). SA-β-gal-positive cells were identified as blue-stained cells under a light microscope.

### 2.15. Statistical Analysis

All statistical analyses were performed using GraphPad Prism version 8.0.2. Each experiment was independently repeated at least three times. Data are presented as mean ± standard error of the mean (SEM). For comparisons between two groups, statistical significance was determined using a two-tailed Student’s *t*-test. For comparisons among more than two groups, one-way analysis of variance (ANOVA) was performed, followed by Tukey’s post hoc test. A *p*-value less than 0.05 (*p* < 0.05) was considered statistically significant.

## 3. Results

### 3.1. Construction of SV2C-Overexpressing Lentiviral Vector

**Figure 1:**
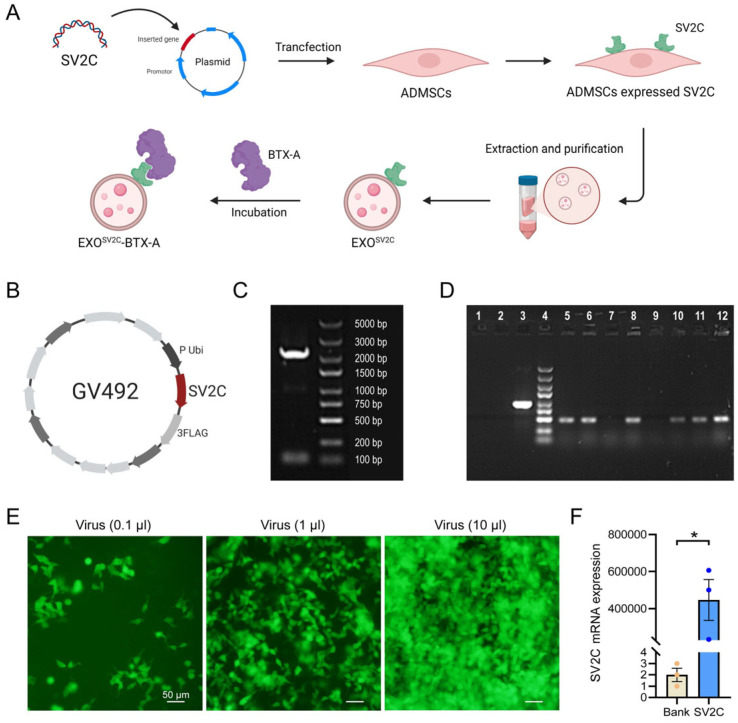
Construction and transfection of SV2C-overexpressing lentiviral vector. (**A**) Schematic diagram illustrating the construction of the exosome–BTX-A complex. (**B**) Schematic representation of the SV2C-overexpressing plasmid; the red segment indicates the SV2C gene insertion site. (**C**) Agarose gel electrophoresis confirmed the SV2C target gene fragment size of 2225 bp, indicating successful plasmid construction. (**D**) PCR analysis of E. coli colony transformation: lane 1, ddH_2_O (negative control); lane 2, empty vector control; lane 3, GAPDH (positive control); lane 4, marker; lanes 5–12, transformed colonies. (**E**) Representative immunofluorescence images showing green fluorescence in HEK293T cells transduced with lentivirus at varying volumes, indicating successful transfection (bar = 50 μm). (**F**) Relative SV2C mRNA expression in HEK293T cells before and after lentiviral transduction, measured by RT-PCR (*n* = 3). Data are presented as mean ± SEM. * *p* < 0.01.

To obtain exosomes capable of binding BTX-A via SV2C-mediated interactions, we aimed to construct exosomes with high SV2C expression on their surface (Figure 1A). We first designed and constructed the GV492-SV2C-GFP recombinant plasmid (Figure 1B). Agarose gel electrophoresis confirmed that the SV2C band was 2225 bp, consistent with the expected size, verifying successful plasmid construction (Figure 1C). Colony PCR further confirmed positive transformants (476 bp, samples 5–12) (Figure 1D). Transfection of the SV2C plasmid into HEK293T cells resulted in green fluorescence, indicating successful transfection (Figure 1E). Fluorescence-based quantification showed a maximum viral titer of 2.5 × 10^8^ TU/mL. qPCR analysis revealed significantly elevated SV2C expression in transfected HEK293T cells compared to controls (Figure 1F). These results demonstrate that the SV2C-overexpressing lentiviral vector was successfully constructed and efficiently transduced HEK293T cells.

### 3.2. Generation of SV2C-Overexpressing ADMSCs

**Figure 2:**
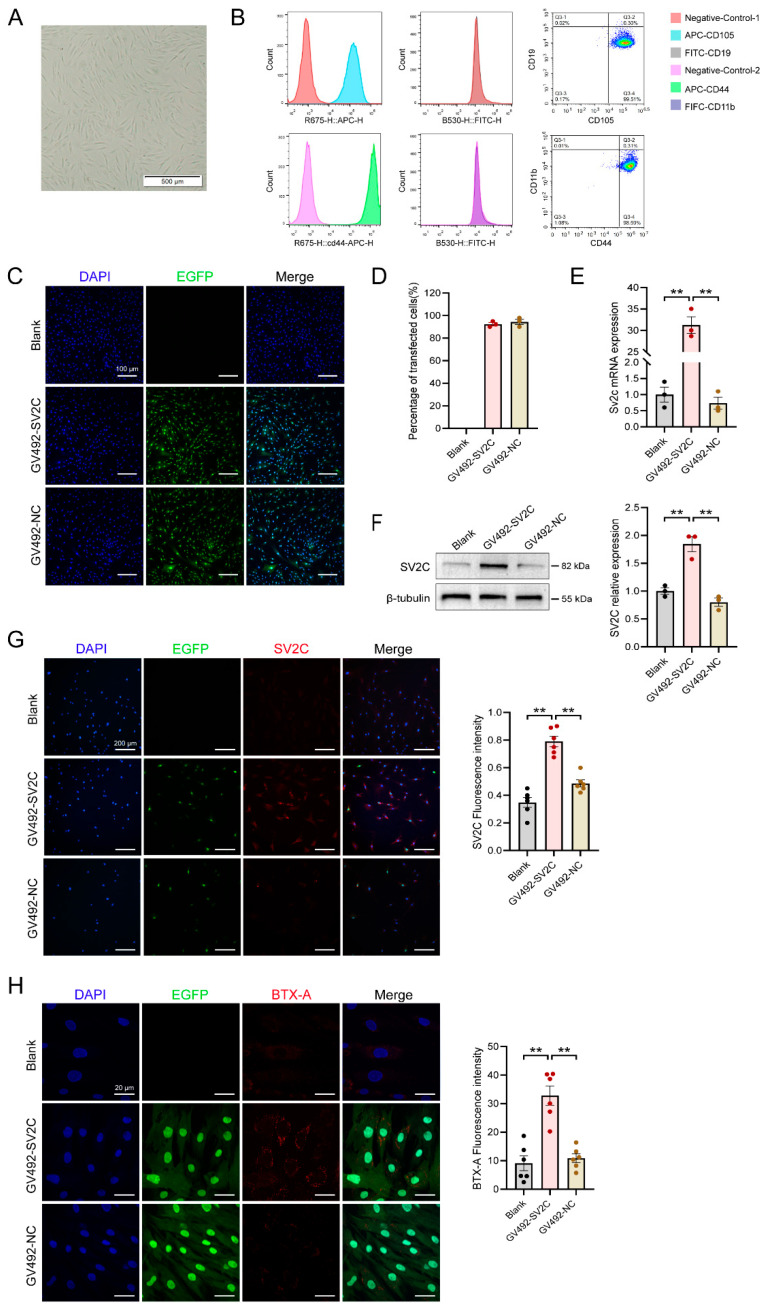
Generation and characterization of SV2C-overexpressing ADMSCs. (**A**) Morphology of cultured ADMSCs under a light microscope, showing typical spindle-shaped cells arranged in parallel, consistent with the characteristic morphology of ADMSC (bar = 500 μm). (**B**) Flow cytometric analysis of surface markers: ADMSCs were positive for CD105 and CD44 (APC fluorescence) and negative for CD19 and CD11b (FITC fluorescence), confirming the mesenchymal phenotype. (**C**) Representative immunofluorescence images of ADMSCs transduced with SV2C lentivirus, showing EGFP (green) and DAPI-stained nuclei (blue) (bar = 100 μm). (**D**) Percentage of EGFP-positive cells after puromycin selection (*n* = 3). (**E**) Relative SV2C mRNA expression levels in blank control, SV2C-overexpressing, and empty vector groups, quantified by qPCR (*n* = 3). (**F**) Representative Western blot image (left) and densitometric quantification (right) of SV2C protein expression in ADMSCs from the three groups (*n* = 3). (**G**) Representative immunofluorescence images showing SV2C (red), EGFP (green), and DAPI (blue) in each group, along with quantitative analysis (bar = 200 μm, *n* = 3). (**H**) Representative images and quantification of BTX-A (red) binding in each group of cells, assessed by immunofluorescence (bar = 20 μm, *n* = 3). Data are presented as mean ± SEM. ** *p* < 0.01.

Primary-cultured human ADMSCs exhibited a spindle-shaped morphology and orderly alignment, consistent with the characteristics of mesenchymal stem cells (Figure 2A). Flow cytometry showed that 99.51% of cells were CD105-positive and CD19-negative, while 98.59% were CD44-positive and CD11b-negative, confirming the identity of ADMSCs (Figure 2B). Following lentiviral transduction with SV2C, cells displayed green fluorescence under microscopy, whereas untransduced controls did not, indicating successful transduction (Figure 2C). After puromycin selection and two passages, purified SV2C-overexpressing ADMSCs were obtained (Figure 2D). RT-PCR and Western blotting confirmed significantly elevated SV2C mRNA and protein levels in the transduced ADMSCs (Figure 2E,F). Immunofluorescence staining further confirmed robust intracellular SV2C expression in the SV2C group compared to the empty vector and blank controls (Figure 2G). Following incubation with 3 U/mL BTX-A, immunofluorescence analysis demonstrated that SV2C-overexpressing ADMSCs bound BTX-A more effectively than low-expressing controls (Figure 2H). These findings confirm the successful generation of SV2C-overexpressing ADMSCs and their enhanced binding capability to BTX-A.

### 3.3. SV2C-Exosomes Bind BTX-A and Reduce Cytotoxicity

**Figure 3:**
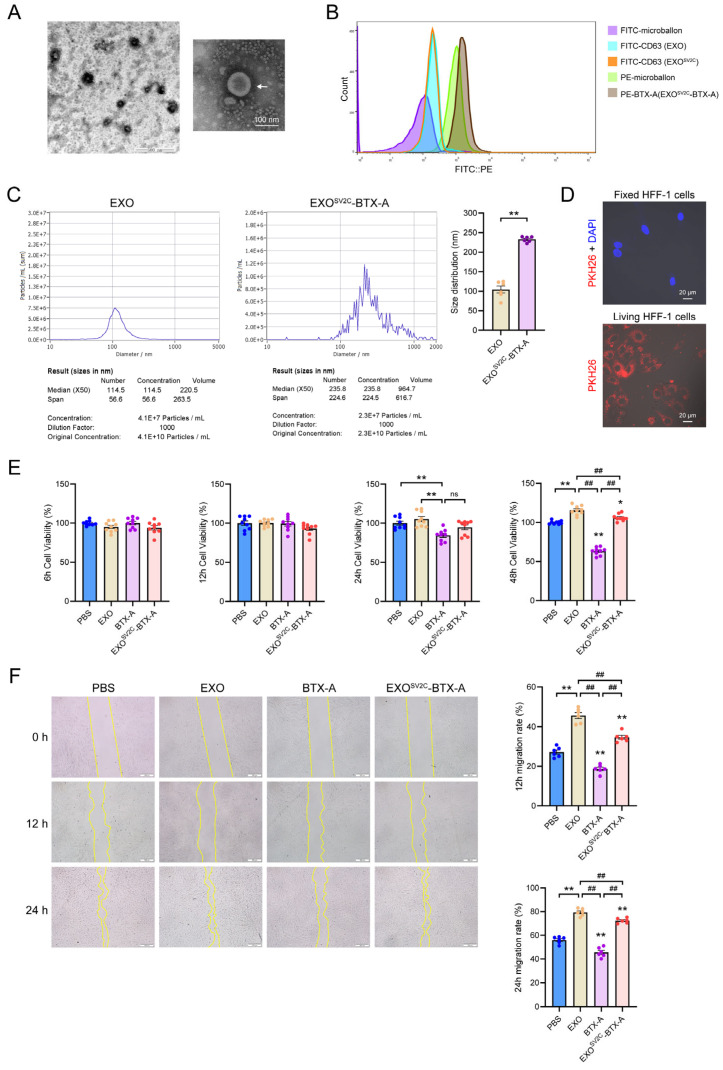
Preparation and characterization of exosomes and EXO^SV2C^-BTX-A complexes. (**A**) Transmission electron microscopy images of purified exosomes isolated from ADMSCs, showing typical round membrane-bound morphology (bar = 500 nm). (**B**) Flow cytometry detection of CD63 and BTX-A on exosomes in EXO and EXO^SV2C^-BTX-A groups using microsphere-conjugated antibodies. (**C**) Nanoparticle tracking analysis of particle size distribution: median size of EXO = 118.6 nm, mean = 122.0 ± 2.96 nm; EXO^SV2C^-BTX-A = 235.8 nm (median), mean = 235.2 ± 5.08 nm (*n* = 6). (**D**) Immunofluorescence images of HFF-1 cells incubated with PKH26-labeled exosomes (red) for 2 h. DAPI-stained nuclei are shown in blue, confirming uptake by both live and fixed cells (bar = 20 μm). (**E**) CCK-8 assay evaluating the viability of HFF-1 cells treated with EXO, BTX-A, or EXO^SV2C^-BTX-A (*n* = 9). (**F**) Scratch wound assay assessing the effects of EXO, BTX-A, or EXO^SV2C^-BTX-A on HFF-1 cell migration (bar = 500 μm, *n* = 6). Data are presented as mean ± SEM. * *p* < 0.05, ** *p* < 0.01 vs. PBS group; ^##^ *p* < 0.01; ns, no significant.

To generate an efficient exosome–toxin complex, engineered exosomes were isolated from SV2C-overexpressing ADMSCs using ultrafiltration and differential centrifugation. Transmission electron microscopy revealed cup-shaped membrane vesicles exhibiting characteristic exosomal morphology (Figure 3A). After co-incubation with 3 U/mL BTX-A for 1 h, the resulting EXO^SV2C^-BTX-A complex was analyzed using bead-assisted flow cytometry. Due to the nanoscale size of exosomes, conventional flow cytometry is insufficient; therefore, fluorescent bead-based capture technology was employed to overcome this limitation. FITC-labeled antibodies were used to detect CD63, and PE-labeled antibodies were used to detect BTX-A, with FITC and PE fluorescence intensities reflecting CD63 expression and BTX-A content, respectively. EXO^SV2C^-BTX-A exhibited significantly higher PE fluorescence than the EXO group, indicating enhanced BTX-A binding, while CD63 levels were comparable between the two groups, suggesting similar exosome quantities (Figure 3B). Nanoparticle tracking analysis showed mean diameters of 122 ± 2.96 nm (median: 118.6 nm) for EXO and 235.2 ± 5.08 nm (median: 235.8 nm) for EXO^SV2C^-BTX-A, indicating that SV2C overexpression and BTX-A binding increased exosomal size (Figure 3C). Cellular uptake assays demonstrated that PKH26-labeled exosomes were efficiently internalized by live HFF-1 fibroblasts, as indicated by the presence of red fluorescence after 2 h of co-culture. No fluorescence was observed in fixed HFF-1 cells, suggesting active uptake by viable cells rather than passive binding (Figure 3D). CCK-8 assays revealed that EXO treatment enhanced the viability and proliferation of HFF-1 cell. Although proliferation in the EXO^SV2C^-BTX-A group was slightly reduced compared to EXO alone, it remained significantly higher than the BTX-A group, indicating that exosome encapsulation alleviated BTX-A-induced cytotoxicity (Figure 3E). Similarly, scratch assays indicated that EXO^SV2C^-BTX-A enhanced fibroblast migration compared to BTX-A alone (Figure 3F). Collectively, these findings demonstrate that SV2C-expressing exosomes can effectively bind BTX-A, reduce its cytotoxicity, and enhance the compatibility of fibroblasts.

### 3.4. EXO^SV2C^-BTX-A Inhibits D-Galactose-Induced Collagen Loss and Cellular Senescence

**Figure 4:**
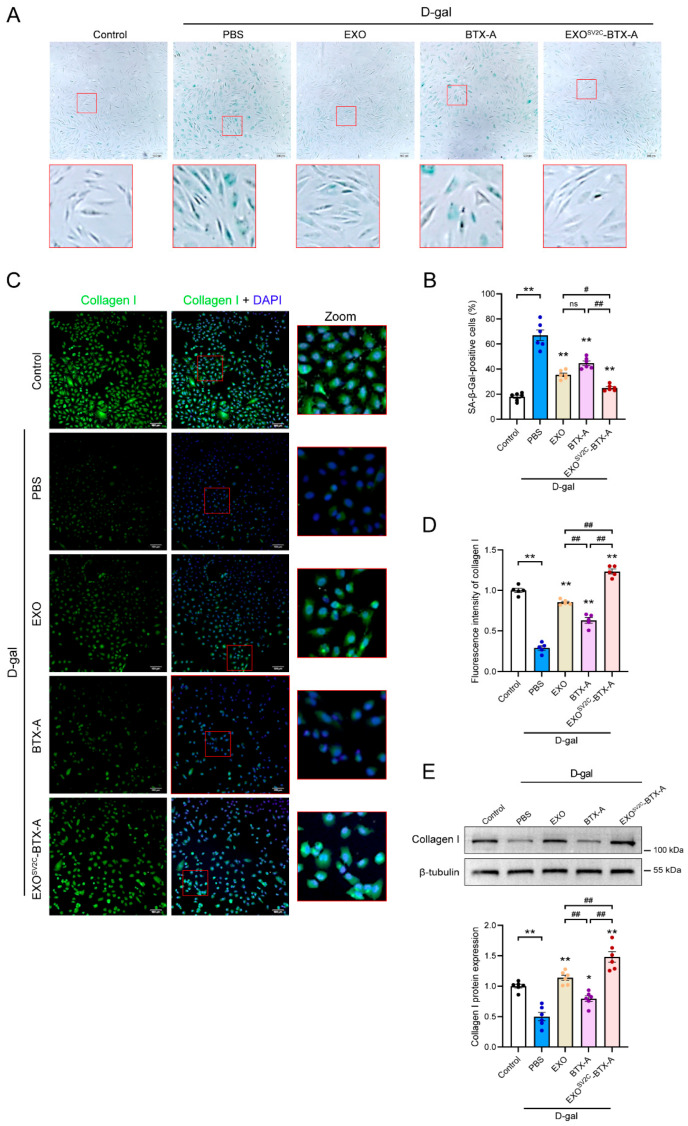
Effects of exosome–BTX-A complexes on D-galactose-induced senescence in HFF-1 cells. (**A**) Representative images of SA-β-gal staining in HFF-1 cells following D-galactose exposure and subsequent treatment with EXO, BTX-A, or EXO^SV2C^-BTX-A (bar = 200 μm). (**B**) Quantification of SA-β-gal-positive cells in each treatment group (*n* = 6). (**C**) Representative immunofluorescence images showing type I collagen expression in HFF-1 cells from each group (bar = 100 μm). (**D**) Quantitative analysis of collagen type I immunofluorescence intensity in each group (*n* = 5). (**E**) Representative Western blot image and quantification of type I collagen expression (*n* = 6). Data are presented as mean ± SEM. * *p* < 0.05, ** *p* < 0.01 vs. D-gal + PBS group; ^#^ *p* < 0.05, ^##^ *p* < 0.01; ns, no significant.

D-galactose (D-gal) is a commonly used inducer of cellular aging, known to accelerate senescence through oxidative stress and the accumulation of advanced glycation end products (AGEs) [30,31]. In this study, senescence of HFF-1 cells induced by D-gal was confirmed via β-Galactosidase staining. Treatment with EXO, BTX-A, or EXO^SV2C^-BTX-A significantly reduced the number of senescent cells (Figure 4A), with the EXO^SV2C^-BTX-A group showing a significantly lower proportion of senescent cells compared to both the EXO and BTX-A groups (Figure 4B). Type I collagen accounts for approximately 80% of total human collagen, and its reduction is a key biomarker of skin aging [31]. Immunofluorescence analysis revealed that D-gal treatment substantially suppressed type I collagen expression in HFF-1 cells, while all three interventions—EXO, BTX-A, and EXO^SV2C^-BTX-A—effectively alleviated collagen degradation and promoted collagen synthesis (Figure 4C,D). Quantitative protein analysis further confirmed that EXO^SV2C^-BTX-A treatment resulted in the highest type I collagen levels, suggesting superior synergistic anti-aging efficacy (Figure 4E). Taken together, these results suggest that EXO^SV2C^-BTX-A significantly suppresses D-gal-induced cellular senescence and promotes type I collagen synthesis.

### 3.5. EXO^SV2C^-BTX-A Attenuates D-Galactose-Induced Skin Aging in Mice

**Figure 5:**
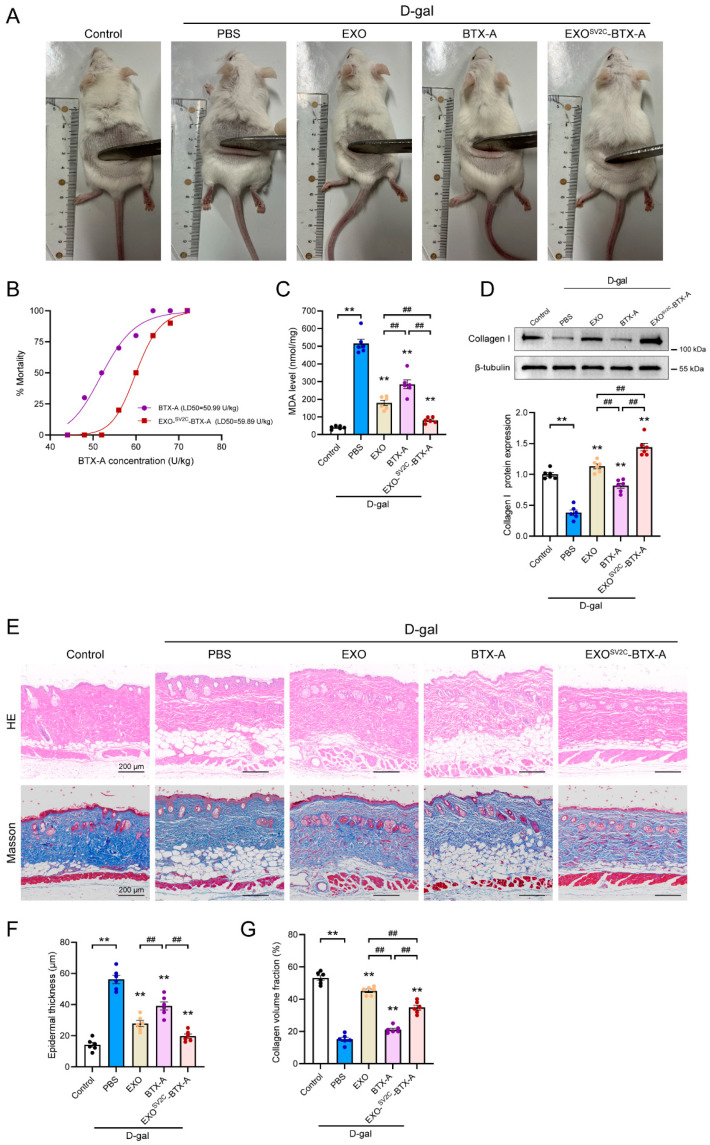
Effects of exosome–BTX-A complexes on D-galactose-induced skin aging in mice. (**A**) Representative images of mouse skin conditions following 42 days of D-galactose administration in different treatment groups. (**B**) Mortality curves and calculated LD_50_ values of mice treated with increasing doses of BTX-A. (**C**) Quantification of malondialdehyde (MDA) levels in skin tissues of each group (*n* = 6). (**D**) Representative Western blot images and quantification of type I collagen protein levels in mouse skin (*n* = 6). (**E**) Representative images of hematoxylin and eosin (H&E) and Masson’s trichrome staining of mouse skin sections. Red: muscle fibers; blue: collagen fibers (bar = 200 μm). (**F**) Quantification of epidermal thickness in each group (*n* = 6). (**G**) Percentage area of collagen fibers in the epidermis (*n* = 6). Data are presented as mean ± SEM. ** *p* < 0.01 vs. D-gal+PBS group; ^##^ *p* < 0.01.

To further assess the anti-aging effects of EXO^SV2C^-BTX-A in vivo, we evaluated skin appearance in mice induced to age by D-gal. Normal mice exhibited smooth, taut skin and dense, glossy fur, whereas D-gal-treated mice exhibited hair thinning, reduced skin elasticity, and dermal sagging (Figure 5A). Treatment with EXO, BTX-A, or EXO^SV2C^-BTX-A led to visible improvements in skin texture and fur condition. However, high-dose BTX-A and EXO^SV2C^-BTX-A induced toxicity (e.g., “wasp waist”). To determine whether exosomal encapsulation alleviated BTX-A toxicity, we conducted a dose-escalation lethality assay. The calculated LD_50_ for the EXO^SV2C^-BTX-A group was 59.89 U/kg, which was higher than the BTX-A group (50.99 U/kg), and also exceeded the safety threshold of 44 U/kg for BTX-A (Figure 5B), indicating reduced acute toxicity via exosomal encapsulation. In terms of safety, no gross pathological abnormalities were observed in the liver, lung, or kidney of normal mice 4 days after treatment with EXO, BTX-A, or EXO^SV2C^-BTX-A (Figure S1A). However, hematological analysis revealed that BTX-A significantly reduced white blood cells, lymphocytes, and red blood cells, as well as hemoglobin, while neutrophils were elevated, indicating systemic toxicity. In contrast, the EXO^SV2C^-BTX-A group showed no such abnormalities, and values remained close to normal ranges (Figure S1B). Serum biochemical analysis further demonstrated that BTX-A elevated liver enzymes such as alanine aminotransferase (ALT) and aspartate aminotransferase (AST), while EXO^SV2C^-BTX-A-treated mice showed better liver safety in these parameters (Figure S1C). In D-gal-induced aging mice, D-gal treatment itself did not significantly disrupt most hematological or biochemical parameters, except for a mild reduction in platelet count and an elevated AST level, indicating a subtle trend towards hepatic toxicity. Neither EXO nor BTX-A exacerbated these changes, suggesting acceptable systemic tolerance (Figure S2). MDA levels in skin tissues—a marker of oxidative stress—were significantly reduced by all treatments, with the lowest level observed in the EXO^SV2C^-BTX-A group (Figure 5C). Type I collagen content was significantly higher in the EXO and EXO^SV2C^-BTX-A groups compared to the D-gal+PBS group (Figure 5D). HE staining showed epidermal thickening, keratinization, and reduced dermal appendages in D-gal mice, which were attenuated by EXO and EXO^SV2C^-BTX-A treatment (Figure 5E,F). Masson staining confirmed that EXO^SV2C^-BTX-A prevented collagen degradation and improved fiber alignment (Figure 5G). These findings indicate that EXO^SV2C^-BTX-A alleviates D-gal-induced skin aging by reducing oxidative stress, enhancing collagen synthesis, and preserving skin structure, while offering superior systemic safety and lower toxicity compared to free BTX-A.

## 4. Discussion

In this study, we successfully engineered ADMSCs stably overexpressing SV2C, a known receptor of BTX-A, and successfully generated SV2C-enriched functional exosomes. Upon co-incubation with BTX-A, a structurally stable EXO^SV2C^-BTX-A complex was formed. This complex exhibited significantly lower toxicity than free BTX-A in both in vitro and in vivo settings, while maintaining superior efficacy in promoting fibroblast migration and type I collagen synthesis. These findings support the potential of exosomal encapsulation strategies in enhancing the safety and therapeutic index of BTX-A.

Previous studies have demonstrated that BTX-B selectively binds to PC12 cells that highly express its receptor, synaptotagmin 2 (SYT2), but fails to bind SYT2-deficient cells [32]. Engineered exosomes expressing SYT2/ganglioside GT1b on their surfaces have also been shown to form high-affinity complexes with BTX-B [33]. Inspired by these findings, we introduced SV2C into ADMSCs and confirmed that both the engineered cells and their secreted exosomes efficiently bound BTX-A. To overcome the detection limitations of nanoscale vesicles in traditional flow cytometry, we developed a bead-assisted flow cytometry method using CD63/BTX-A antibody-coated microspheres, enabling the simultaneous detection of CD63 and BTX-A fluorescence. The successful dual labeling confirmed efficient and stable complex formation. Notably, CD63 levels remained similar between EXO and EXO^SV2C^-BTX-A groups, indicating comparable exosome quantities, while BTX-A signals were significantly enriched in EXO^SV2C^-BTX-A, confirming the SV2C-enhanced binding affinity.

Although BTX-A is widely used in scar treatment and aesthetic medicine, its cytotoxicity toward fibroblasts limits its use in tissue regeneration and anti-aging applications. Studies have shown that BTX-A inhibits the proliferation and migration of human skin fibroblasts [15] and modulates fibrosis during wound healing, thereby reducing scarring [34]. In our study, free BTX-A significantly suppressed fibroblast viability and migration, whereas EXO^SV2C^-BTX-A reversed these inhibitory effects, highlighting the dual function of SV2C-exosomes in both detoxification and functional enhancement. Both CCK-8 and scratch assays supported that EXO^SV2C^-BTX-A enhanced fibroblast proliferation and migration more effectively than exosomes or BTX-A alone, suggesting synergistic effects in tissue repair and cytoprotection.

Building on the cellular assays, we employed D-galactose-induced models of fibroblast senescence and murine skin aging to assess the anti-aging potential of EXO^SV2C^-BTX-A comprehensively. D-galactose induces senescence through the accumulation of advanced glycation end-products (AGEs), structural disruption of collagen and elastin, and the induction of oxidative stress, thereby mimicking natural aging processes [35,36,37]. Prior studies have shown that exosomes alleviate cellular aging by downregulating β-galactosidase and matrix metalloproteinase (MMP)-1 and MMP-3 expression while increasing type I collagen synthesis in HSF [38,39,40,41]. Consistently, our study found that EXO^SV2C^-BTX-A markedly decreased senescence-associated β-galactosidase levels and enhanced type I collagen production, surpassing the individual effects of EXO or BTX-A, indicating a synergistic anti-senescence benefit. In vivo, EXO^SV2C^-BTX-A significantly reduced MDA levels, preserved sebaceous glands and hair follicles, reduced epidermal keratinization, and enhanced dermal collagen deposition, thus reversing D-galactose-induced skin aging. Importantly, toxicity evaluation showed that the LD_50_ of EXO^SV2C^-BTX-A was higher than that of free BTX-A in mice, indicating improved biosafety. This is particularly relevant for the use of chronic or repeated BTX-A in clinical anti-aging interventions or chronic wound healing.

Nonetheless, several limitations remain. First, the binding ratio between exosomes and BTX-A has not been precisely defined, and the binding efficiency and purity of the complex remain undefined. Although we attempted to quantify this interaction using centrifugation-coupled ELISA, the sensitivity was suboptimal. Future studies may employ more accurate techniques, such as liquid chromatography or surface plasmon resonance, for more in-depth analysis. Second, the optimal loading ratio of EXO^SV2C^ to BTX-A has yet to be determined. Dose-gradient experiments will be considered to evaluate synergistic effects and functional dependency. Moreover, the molecular mechanisms underlying the observed anti-aging effects—particularly those involving collagen biosynthesis—warrant deeper exploration. The current regimen of daily injections, though effective, also poses practical limitations for clinical application. In future work, in vivo imaging with fluorescent or radiolabel tracers may help determine biodistribution and tissue retention, thereby informing optimized dosing schedules. In conclusion, we developed a novel SV2C-functionalized exosome platform that enables efficient BTX-A loading, reduces its acute toxicity, and enhances anti-aging and tissue repair outcomes in cellular and animal models.

## 5. Conclusions

In this study, we successfully constructed SV2C-overexpressing ADMSCs that secrete functionalized exosomes capable of binding BTX-A through in vitro co-incubation, resulting in a stable EXO^SV2C^-BTX-A complex. This complex retained BTX-A bioactivity while significantly reducing its cytotoxicity in vitro and in vivo, enhancing cell viability and migration. In D-galactose-induced aging models of both fibroblasts and murine skin, EXO^SV2C^-BTX-A demonstrated superior anti-aging effects compared to BTX-A or exosomes alone. Collectively, these findings suggest that SV2C-functionalized exosomes hold promise for enhancing BTX-A therapeutic efficacy, reducing toxicity and expanding its clinical applications in skin rejuvenation, scar repair, and toxicity mitigation.

## Data Availability

The data that support the findings of this study are available from the corresponding author upon reasonable request.

## Supplementary Materials

The following supporting information can be downloaded at: https://www.mdpi.com/article/10.3390/biology14081040/s1, Figure S1: Organ morphological and hematological/biochemical evaluation following treatment in normal mice; Figure S2: Hematological and biochemical evaluation in D-galactose-induced aging mice. Supplemental Materials—Original western blot images.

## Abbreviations

The following abbreviations are used in this manuscript:

BTX-A	Botulinum toxin type A
SV2C	synaptic vesicle glycoprotein 2C
EXO^SV2C^	SV2C-overexpressing exosomes
MSCs	Mesenchymal stem cells
MDA	Malondialdehyde
