# Supplementary material for: Engineered Exosomes Complexed with Botulinum Toxin Type A for Enhanced Anti-Aging Effects on Skin
Source: Biology (Basel). 2025 Aug 13;14(8):1040. doi: 10.3390/biology14081040 (PMC12383945; doi:10.3390/biology14081040)
Supplement: Supplementary file 1 [file biology-14-01040-s001.zip › biology-3751804-supplementary.pdf]

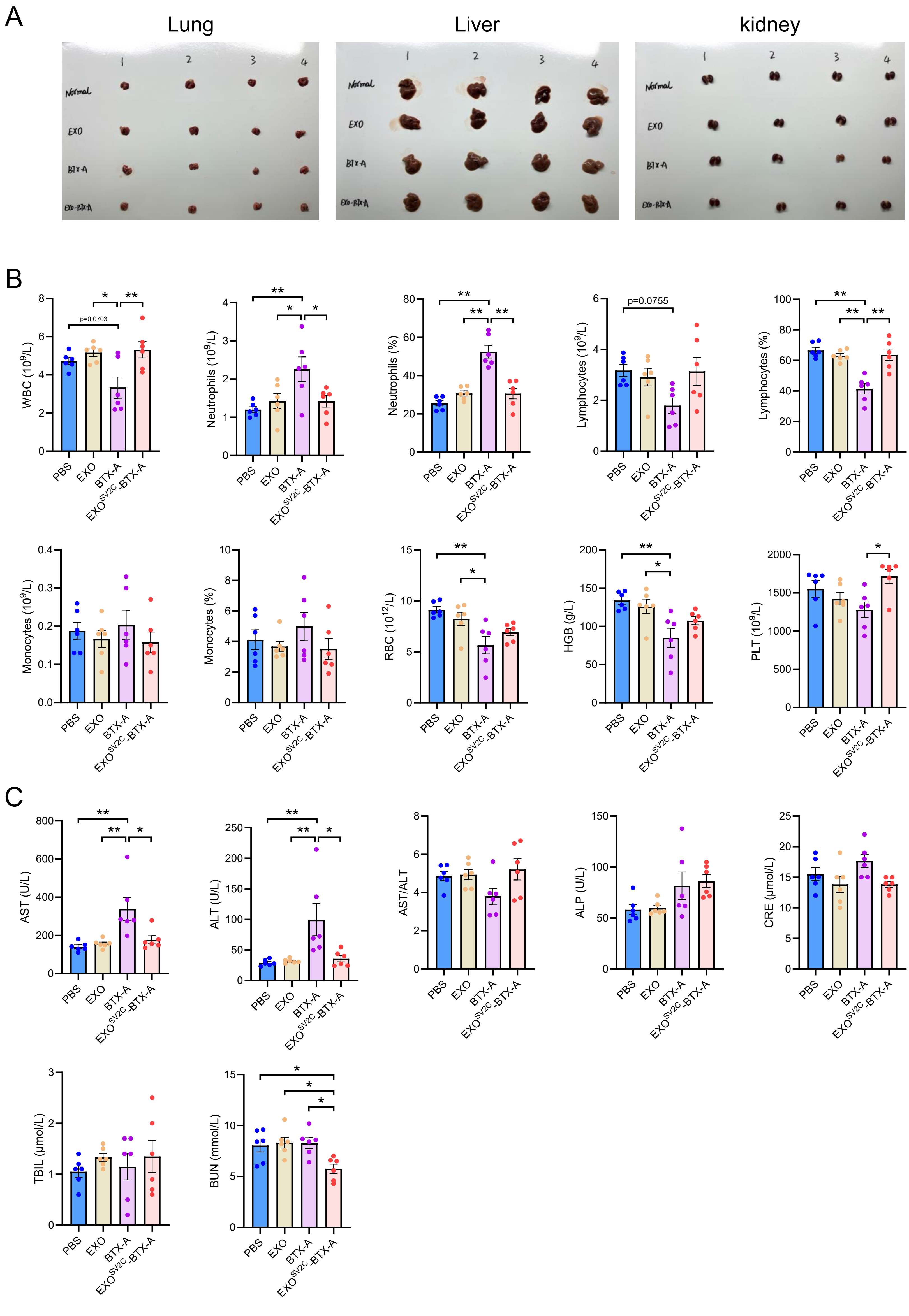

Supplementary Figure S1. Organ morphological and hematological/biochemical evaluation following treatment in normal mice. (A) Representative images of the lung, liver, and kidney harvested from normal mice 4 days after intraperitoneal injection of EXO (2.0×10^7 particles/mL), BTX-A (50.99 U/kg), or EXO^{SV2C}-BTX-A (2.0×10^7 particles/mL containing 50.99 U/kg BTX-A). No evident morphological abnormalities were observed in any of the groups. (B) Hematological analysis of peripheral blood. WBC, white blood cells; RBC, red blood cells; HGB, hemoglobin; PLT, platelets. (C) Serum biochemical analysis of liver and kidney function. AST, aspartate aminotransferase; ALT, alanine aminotransferase; ALP, alkaline phosphatase; CRE, creatinine; TRIL, total bilirubin; BUN, blood urea nitrogen. Data are presented as mean ± SEM (n = 6 per group). *$p < 0.05$, **$p < 0.01$.

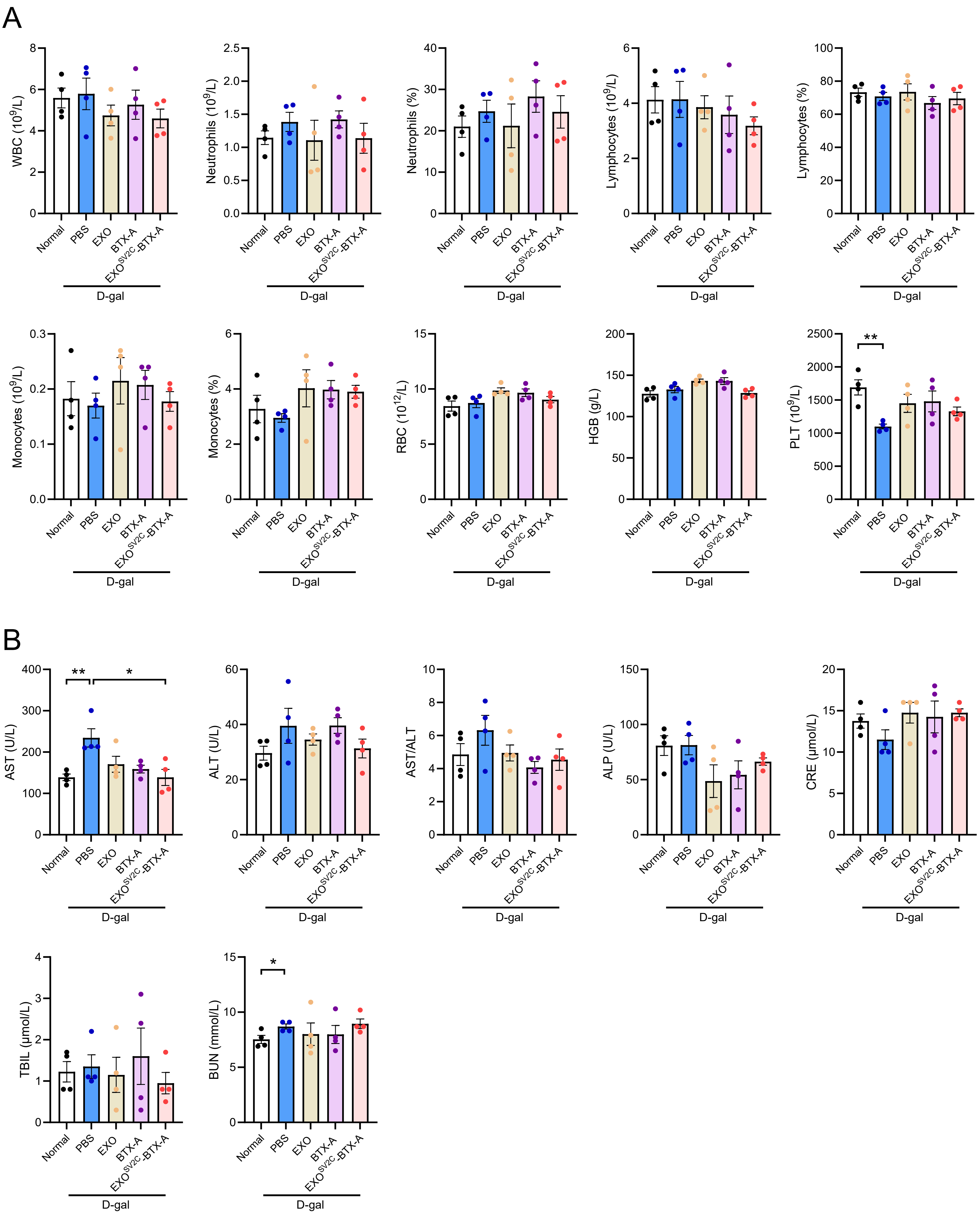

Supplementary Figure S2. Hematological and biochemical evaluation in D-galactose-induced aging mice. (A) Hematology analysis of peripheral blood from D-galactose (1000 mg/kg)-induced aging model mice treated with EXO (2.0×10^7 particles/mL), BTX-A (3.0 U/ml), or EXO^{SV2C}-BTX-A (2.0×10^7 particles/mL, 3.0 U/ml BTX-A). WBC, white blood cells; RBC, red blood cells; HGB, hemoglobin; PLT, platelets. (B) Serum biochemical analysis. AST, aspartate aminotransferase; ALT, alanine aminotransferase; ALP, alkaline phosphatase; CRE, creatinine; TRIL, total bilirubin; BUN, blood urea nitrogen. Data are presented as mean ± SEM (n = 4 per group). *$p < 0.05$, **$p < 0.01$.
