# Supplementary figures and images for: Engineered Exosomes Complexed with Botulinum Toxin Type A for Enhanced Anti-Aging Effects on Skin
Source: Biology (Basel). 2025 Aug 13;14(8):1040. doi: 10.3390/biology14081040 (PMC12383945; doi:10.3390/biology14081040)

Supplemental Materials -Original western blot images

Figure.2F

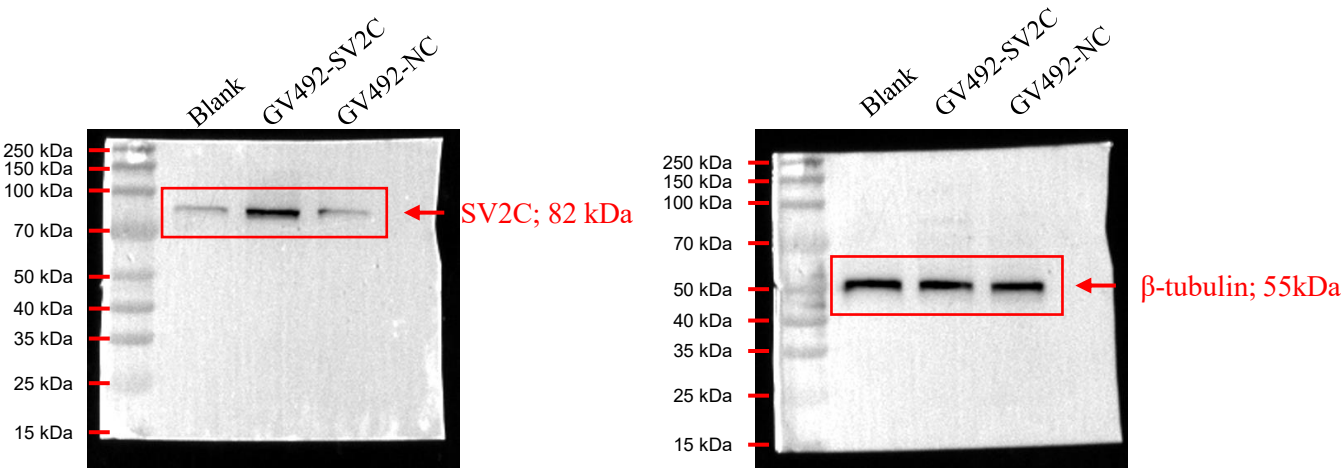

Figure.4E

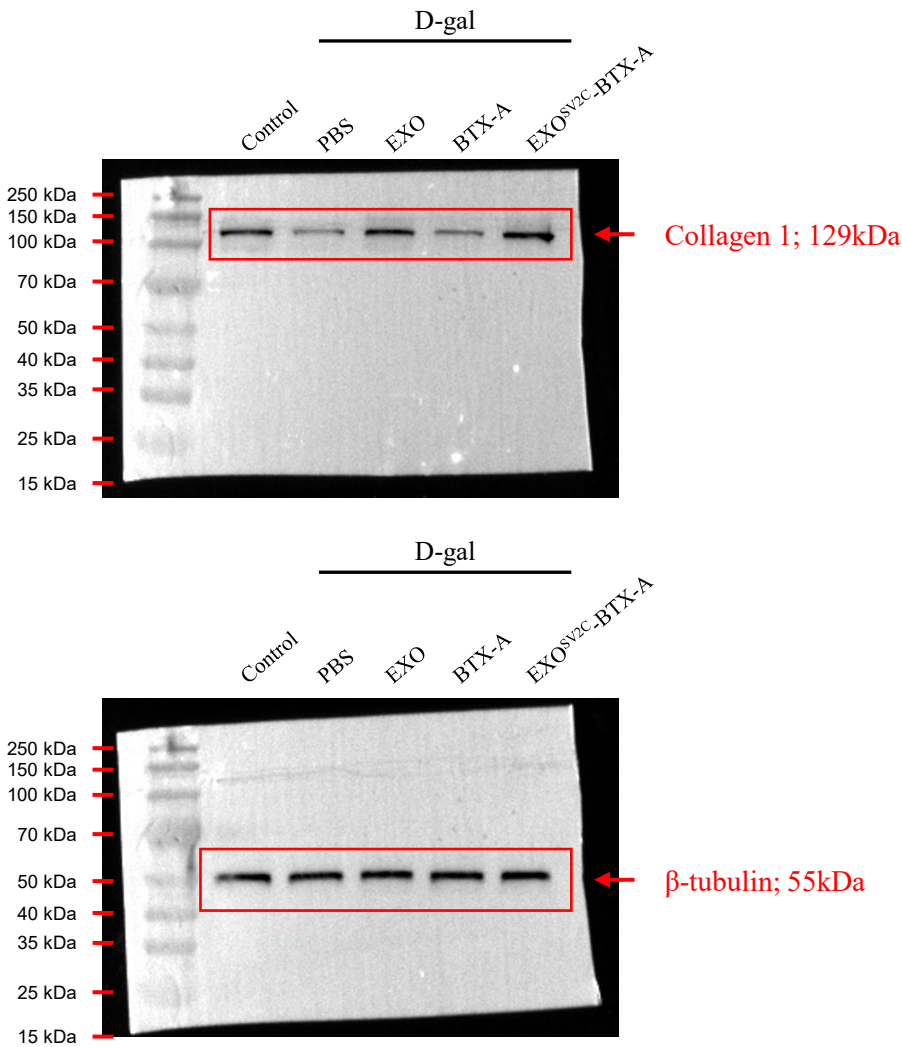

Figure.5D

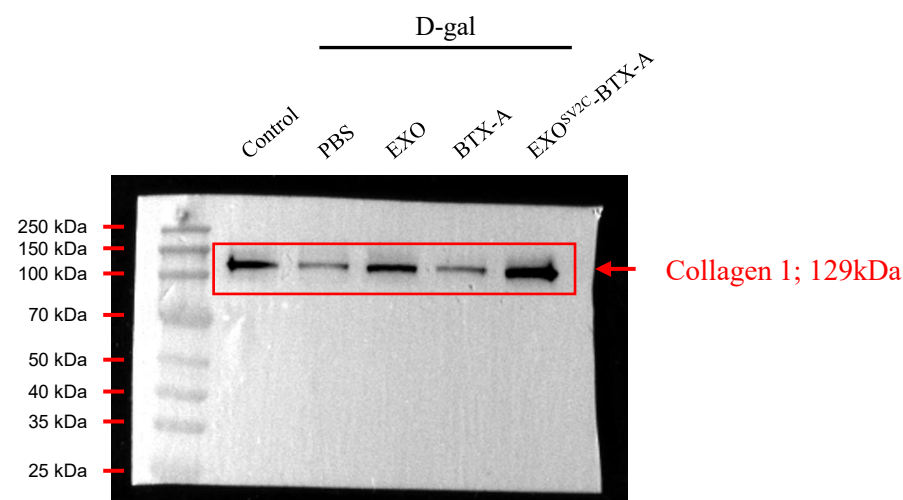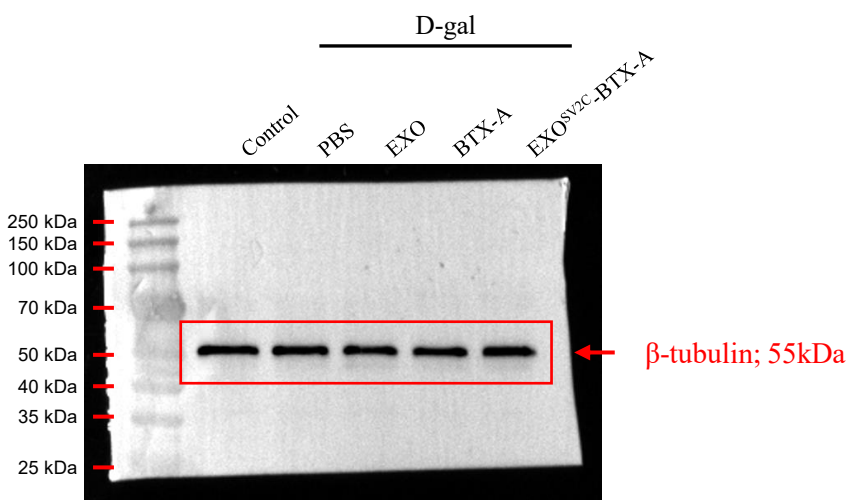

Supplement: Supplementary file 1 [file biology-14-01040-s001.zip › biology-3751804-Original western blot images 8-8 .pdf]
